# The impact of digital economy on regional technological innovation capability: An analysis based on China’s provincial panel data

**DOI:** 10.1371/journal.pone.0288065

**Published:** 2023-07-14

**Authors:** Zhenxing Tian, Ying Li, Xiaogeng Niu, Meiyu Liu

**Affiliations:** 1 School of Economics, Hebei GEO University, Shijiazhuang, China; 2 Natural Resource Asset Capital Research Center, Hebei GEO University, Shijiazhuang, Hebei, China; Shenzhen University, CHINA

## Abstract

The development of the digital economy in China facilitates the transformation of old and new growth drivers. It can greatly promote the upgrading of technological innovation capacity, realize economies of scale and scope, and constantly promote the generation of new industries and new forms of business with the deep integration of digital economy and traditional industries, thereby promoting the high-quality development of China’s economy. This paper uses inter-provincial panel data from 2013 to 2020 and a dynamic spatial Durbin model to quantify the impact of the digital economy on regional technological innovation capability (RTIC). The results show that: (1) the digital economy has positive spatial spillover effect and can boost the province’s and neighboring provinces’ regional technological innovation capability; (2) regional technological innovation capability has obvious spatial and temporal aggregation effects; (3) the impact of the digital economy on RTIC is mainly short-term effects, and there is regional heterogeneity, with the western region experiencing the highest effects and the eastern region experiencing less. Therefore, it is urgent to accelerate the development speed of the digital economy, grasp the law of dynamic economic development, identify the regional heterogeneity of the digital economy development, and deepen inter-regional digital technology cooperation to comprehensively drive the improvement of regional technological innovation capability.

## Introduction

Innovation is the primary driving force for development. Since the beginning of reform and opening-up, China has attached great importance to scientific and technological innovation, and given top priority to innovation in the innovation-driven development strategy and new development paradigms that it has put forth. However, compared with other developed countries, China’s innovation capacity is still at a lower level. To achieve higher quality development and improve international competitiveness, we urgently need to develop new drivers that can stimulate the continuous improvement of innovation capacity. The growth of the digital economy has given us a new avenue for innovative development. On the one hand, the digital economy has the potential to be deeply integrated with traditional industries. It can constantly drive the transformation and upgrading of traditional industries and promote the improvement of their innovative ability relying on data, information, and other emerging production factors, as well as integrating digital technology and other emerging technological means. On the other hand, the digital economy can effectively break geographical boundaries and strengthen the correlation and interactivity of innovation output between regions, thus driving the improvement of the overall innovation capacity between regions. The scale of the digital economy is very huge in China. White Paper on the Development of China’s Digital Economy showed that the scale of China’s digital economy value added expanded from 2.6 trillion yuan in 2005 to 45.5 trillion yuan in 2021, and the proportion of GDP increased from 14.2% to 39.8%, especially from 2017–2021, the average annual compound growth rate of the digital economy scale reached 13.6%, much higher than the GDP growth rate in the same period, which has become a key driver of economic growth.

The impact of digital technology on the economy is growing rapidly [1]. China’s RTIC has steadily improved, and technological innovation has gradually evolved into a driving force in promoting high-quality economic development [2]. In the context of innovation development strategy, digital technology and real industry are intertwined, and customized production and intelligent manufacturing based on digital technology will encourage enterprises to achieve technological innovation, which is critical for expanding the real industry’s development space. The digital economy enables the actual industry to increase production efficiency and structure, which has far-reaching implications for boosting technical innovation in numerous industries and optimizing regional innovation layout. The application of digital technology and the dissemination of data information, guided by the innovation-driven strategy, are conducive to realizing the new pattern of innovation development in various regions of China, strengthening regional innovation cooperation and connectivity, and promoting the continuous improvement of RTIC. From the standpoint of dynamic spatial effect, and it can be decomposed into short-term effect and long-term effect respectively for analysis [3], this paper investigates the impact of the digital economy on RTIC, analyzes its direct and spillover effects from the short and long term, investigates the mechanism of digital economy’s impact on RTIC, and makes pertinent policy recommendations in order to encourage all regions to share the digital economy’s development dividend, contribute to the developed regions’ leadership role in digital economy and promoting coordinated digital economy development contributes to the long-term improvement of RTIC.

Academics have paid close attention to the digital economy as a new economic form that accelerates the reshaping of economic development governance models. Most of the existing studies focus on the statistical measurement and economic effects of the digital economy. Studies on the statistical measurement of the digital economy are abundant and generally fall into two categories. One method is a direct measurement, which measures the scale of the digital economy’s added value within a defined scope [4]. The other method is indirect measurement, which measures the level of development of the regional digital economy by constructing a comprehensive index system [5].The emergence of digital technology has significantly altered innovation and has a greater impact on value creation [6] and value acquisition [7]. The majority of research on the economic effects of the digital economy is done at the micro level. In theory, the constant integration of the Internet and the industry supports the evolution of innovative forms, and the innovation profit model must be revisited as digitalization progresses [8]. Companies in all industries can be pushed by digital transformation to capture value in many forms and establish new business models and ecosystems [9]. According to empirical study, the digital economy can foster innovation and regional production in the long run [10]. Digital transformation is a key driving force for innovation [11] and is helpful to innovate business models, improve enterprise value [12, 13] and help enterprises gain competitive advantages in the digital economy environment [14]. The use of big data can significantly improve the economic performance of businesses, and big data investment plays an intermediary role [15]. At the macro level, the increased productivity of digital assets results in a significant decrease in employment in mass production systems [16].

A review of the literature reveals that scholars have reached a consensus on the argument that the digital economy will boost RTIC. The emergence and proliferation of digital technologies aim to increase the innovation potential of most organizations [17, 18]. Some scholars have proved from micro-data research that data diversity and speed have a positive impact on enterprise innovation performance [19], and big data technology has an indirect impact on RTIC [20]. According to macroeconomic studies, the digital economy in Asian countries can transform business processes through RTIC [21]. The digital economy can promote China’s green innovation capacity [22]. The improvement of profitability can promote enterprises to carry out innovative production, and the digital economy can also improve enterprises’ innovation input through diffusion effect and evolutionary effect, thereby improving enterprises’ innovation performance [23].

Based on the above literature review, the development of digital economy can indeed promote the improvement of innovation ability. However, on the one hand, most of the existing studies are from the theoretical level, and there are few empirical analyses directly exploring the relationship between the two. At the same time, there are few theoretical analyses on the dynamic effects of the two in theoretical studies. On the other hand, most empirical studies use static spatial econometric models to discuss spatial spillover effect, and most choose to use a simple spatial weight matrix of geographical distance and economic distance, and no papers use dynamic spatial econometric model to analyze the dynamic spatial spillover effect of innovation capability. Therefore, this paper intends to extend the following aspects. Firstly, at the level of the theoretical framework, the influence mechanism of the digital economy on RTIC is sorted out based on the existing theoretical framework, and the spatial spillover effect and dynamic effect of the digital economy on RTIC is explored. Secondly, this paper draws on the technology correlation matrix which can better connect the spatial correlation of innovation capability between regions and more accurate analysis of the spatial spillover effects of the digital economy and innovation capability. Finally, at the level of empirical test, the spatial effect of the digital economy on RTIC is empirically tested by dynamic spatial Durbin model (DSDM) on the basis of full consideration of technological linkages among provinces, while the spatial heterogeneity is fully considered and the samples is divided into different regions for an empirical test.

## Theoretical analysis

The review of the literature reveals that scholars’ research conclusions on the impact of the digital economy on RTIC are essentially consistent, namely that the development of the digital economy can significantly promote the improvement of RTIC. Knowledge is the key input in the innovation process [24]. The digital economy can promote the flow of knowledge within enterprises [25], while knowledge integration and application can effectively promote the increase of the number of innovations [26]. The influence mechanism of the digital economy to promote RTIC can affect enterprises on the micro level, industries at the medium level, and then the overall improvement of RTIC on the macro level. At the level of micro-enterprises, enterprises not only need to consume a lot of time, manpower and capital costs in the innovation process, but also face greater uncertainty and sunk cost risks. For risk-averse enterprises, they are reluctant to take risks to make innovation investments. The high cost of innovation will reduce the enthusiasm of enterprises to innovate and is not conducive to the improvement of RTIC. However, with the advent of the era of the digital economy, the digital economy will effectively reduce replication and transportation costs [27]. Data and information, as emerging factors of production, bring new conditions and means of innovation to the development of enterprises and stimulate the innovation vitality of enterprises. Under the pressure of market and technological competition, enterprises’ digital transformation can effectively improve production efficiency and expand the market competitive advantage [28, 29], among which innovation plays an important positive role in such competitive advantage [30], so enterprises will pay more attention to consumer demand [31] and innovation of production management mode, to promote enterprise innovation and development. Digital technology and real industry continue to deep integrate at the medium-industry level to promote industrial innovation and development. On the one hand, data and information, as the foundation of the digital economy development, are characterized by low cost, reusability and low barriers to entry. This allows data to move naturally and be shared among enterprises, greatly improving the efficiency of using data elements. At the same time, the innovative achievements of enterprises can be directly learned and emulated by other enterprises relying on open platforms such as the Internet, which will rapidly carry out a series of innovation activities. On the other hand, the digital economy accelerates the flow of innovation factors with its high permeability and sharing nature, thus contributing to the innovative development of the whole industry. At the macro level, during the development of the digital economy, digital technology penetrates into all sectors of society, driving the RTIC to improve. With the expansion of enterprise production scale, the marginal cost of enterprises relying on e-commerce and mobile payment will continue to decrease, and the marginal revenue of enterprises will gradually increase, and the effect of scale economy of enterprises is obvious. With the integration of traditional industry and digital technology, industrial boundaries are becoming more blurred, and the entire industry chain and supply chain coordinated innovation development is promoting industrial scale economy effect. The scale effect reduces enterprise production costs, encouraging enterprises to carry out continuous technological innovation and the continuous sharing and utilization of data and other resources, which is conducive to continuous innovation and synergistic development of industries, thereby promoting RTIC improvement.

**Fig 1:**
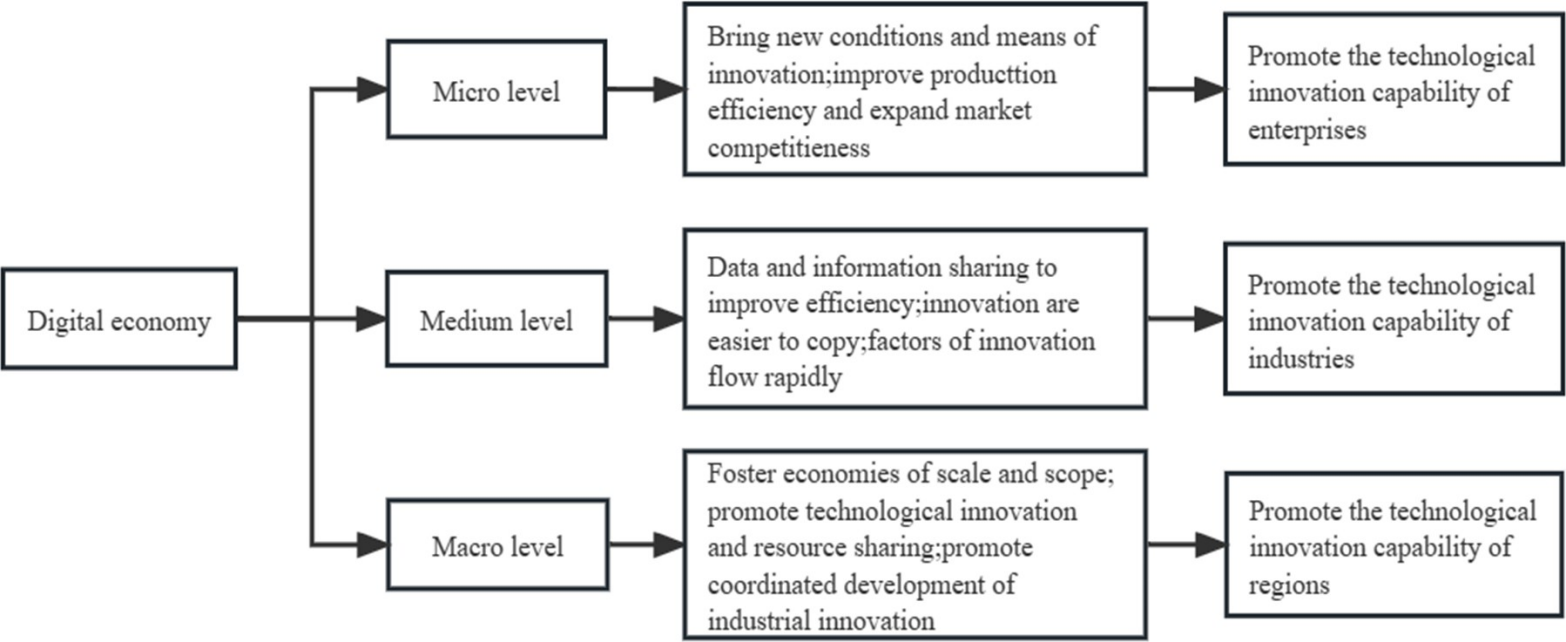
Mechanism of the impact of the digital economy on RTIC.

In conclusion, relying on digital technology to achieve optimal allocation of production factors and maximize resource utilization efficiency, reduce enterprise production costs, can promote enterprise innovation initiative, which in turn promotes technological innovation and digital industrialization, helps optimize industrial layout, and realizes the overall improvement of RTIC. Through empirical research, Xu and Li [32] discovered that the digital economy can foster innovative ability. Thus, hypothesis 1 is proposed in this paper. The mechanisms of these three levels affecting the enhancement of RTIC are shown (see Fig 1).

**Hypothesis 1:** The growth of the digital economy is beneficial to the advancement of RTIC.

The Internet, the Internet of Things, and other platforms are used to diffuse data and information. Digital technology and knowledge have naturally low diffusion costs and high diffusion, and the strong mobility of these data and other emerging production factors makes the inter-regional digital economy more closely connected. With the continuous and extensive diffusion of digital technology and knowledge, innovation participants from different regions can obtain more innovative inspirations and innovative ideas, deepen the learning and exchange among innovation subjects, and promote the generation of new technologies and new production models. With the advancement of modern information networks and communication technology system, resource sharing and knowledge spillover have become an important means of promoting the continuous improvement of neighboring provinces’ technological innovation capacity [33]. In addition, strengthening inter-provincial cooperation and exchanges in the field of digitalization, sharing the fruits and dividends of the development of digital economy, and making innovative digital transformation and upgrading of traditional industries with the help of digital technology can effectively improve the efficiency of data utilization, enhance the concept of enterprise innovation, and improve the efficiency of digital technology upgrading and innovation of enterprises. The spillover and sharing of digital technology and knowledge can promote the improvement of regional innovation output [34]. It will strengthen the positive spillover effect of the digital economy on RTIC, promote the coordinated development of the digital economy and innovation in different regions, and thus can promote the continuous improvement of RTIC. Empirical studies have shown that the digital economy has a large spatial spillover impact on Chinese cities’ innovation capacity, which can not only boost a city’s innovation capacity but also promote the synchronous growth of adjacent cities’ innovation capacity [35]. Based on this, hypothesis 2 is proposed in this paper.

**Hypothesis 2:** The development of the digital economy has a positive spatial spillover effect on the improvement of RTIC in neighboring areas.

The digital economy and RTIC both take a long time to develop and are dynamic developing processes that do not occur overnight. Yu and Liu [36] argue that the innovation output of each stage is at the same time the input of the next stage. Besides, the impact of digital economy development on innovation efficiency exhibits both lag and continuity features [37]. The innovation outcomes and experience gained from the previous innovation inputs can serve as the foundation for new technological breakthroughs later in the process, which can improve the efficiency of technological innovation and thus promote technological innovation and create a favorable innovation environment for enterprises to promote sustainable innovation development in the region. This paper proposes hypothesis 3 based on this.

**Hypothesis 3:** The digital economy promotes the improvement of RTIC in a dynamic development process with time and space lag.

## Variable selection and data sources

### Variable selection

#### Explained variable

Regional technological innovation capability (RTIC). Scholars generally use the number of patent applications or the number of patents granted to measure the level of RTIC in existing studies. The patent application is only a type of protection of the intellectual property of the applicant subject to its innovation, which is not put into use through the certification of professional institutions; whereas the patent grant is the recognition of the state for its innovation achievements, which is considered to have a certain innovation value and can bring benefits to enterprises and promote the development of regional innovation, and most scholars believe that the number of patent grants can more truly and accurately reflect the level of RTIC [38]. As a result, the number of patents granted per 10,000 persons is chosen as the explained variable in this paper to measure the index of RTIC.

#### Explanatory variable

The digital economy (DIEC). There has been a great deal of controversy among scholars regarding the measurement of the digital economy indicators. Inconsistent research results among scholars in this field can be directly attributed to variability in the measurement of digital economy indicators. Comprehensive indicators, as opposed to a single indicator for measuring the digital economy, can more comprehensively and systematically represent the level of the digital economy development. The majority of them are developed in terms of digital infrastructure, scale, and application throughout the existing literature. On the basis of referring to Wang and Chen [39] and considering data integrity and availability, this paper selects 15 secondary indexes to measure 3 primary indexes respectively, and uses entropy method to determine the weights to calculate a comprehensive index for evaluating digital economy. Relevant indexes and their respective weights are shown (see Table 1).

**Table 1:** The digital economy index system and the weight of each sub-index.

First order index	The weight	Secondary indicators	The weight	Direction of influence
Digital infrastructure	0.340 2	Mobile telephone exchange capacity	0.0699	Positive
		Length of long-distance optical cable	0.0710	Positive
		Number of Internet broadband access ports	0.0689	Positive
		Number of domain names	0.0610	Positive
		Number of mobile phone base stations	0.0694	Positive
Level of digital industrialization	0.329 4	Total volume of post and telecommunications services	0.0620	Positive
		Revenue from software business and information technology services	0.0554	Positive
		The coverage breadth of digital finance	0.0723	Positive
		Online mobile payment level	0.0732	Positive
		The proportion of urban unit employees in information technology service industry in urban employment	0.0665	Positive
Level of industry digitization	0.330 4	Number of websites per 100 enterprises	0.0744	Positive
		Digitalization degree of digital finance	0.0721	Positive
		The proportion of enterprises with e-commerce trading activities	0.0607	Positive
		E-commerce sales	0.0717	Positive
		Total amount of express delivery	0.0515	Positive

Note: The level of online mobile payment and the digitalization degree of digital finance are the index data of the coverage breadth of digital finance, the level of online mobile payment and the digitalization degree of digital finance are derived from the Peking University Digital Inclusion Financial Index of the Research Center for Digital Finance of Peking University.

#### Control variables

In addition to the core explanatory variables, this paper selects other control variables that can influence the RTIC. The level of economic development, expressed by real GDP per capita(PGDP); the intensity of foreign direct investment(FDIS), expressed by the total investment of foreign-invested enterprises; the intensity of investment in innovation funds(RDG), expressed by the proportion of R&D investment funds to real GDP; the input of innovation personnel(RDP), expressed by the full-time equivalent of R&D input personnel; the importance of government(GTE), expressed by the proportion of education and science and technology expenditures to general budget expenditures; the level of urbanization(CSL), expressed as the ratio of urban population to year-end population household registration; the degree of intellectual property protection(TEC), expressed as the proportion of technology market turnover to real GDP. The results of descriptive statistics for each indicator are shown (see Table 2).

**Table 2:** Descriptive statistics of each indicator variable.

Variable	Indicator Description	Observation	Mean	Minimum	Maximum	Standard Deviation	Data Sources
*RTIC*	The logarithm of the number of patents granted per 10,000 persons	240	2.012	-0.570	4.309	1.050	China Science and Technology Statistical Yearbook
*DIEC*	The logarithm of digital economy indicators	240	- 1.335	-2.585	-0.247	0.429	*China Statistical Yearbook;* Peking University Digital Inclusion Financial Index of the Research Center for Digital Finance of Peking University
*PGDP*	The logarithm of real GDP per capita	240	1.616	-0.432	2.645	0.412	*China Statistical Yearbook*
*FDIS*	The logarithm of total investment in foreign-invested enterprises	240	4.084	0.614	7.310	1.372	*China Statistical Yearbook*
*RDG*	The logarithm of R&D investment funds as a share of real GDP	240	2.029	-0.914	4.468	1.199	*China Statistical Yearbook;* local statistical bulletins
*RDP*	The logarithm of the full-time equivalent of R&D personnel inputs	240	2.029	-0.914	4.468	1.199	*China Statistical Yearbook;* local statistical bulletins
*GTE*	The logarithm of government spending on science and technology and education as a share of general budget spending	240	2.890	2.339	3.431	0.192	*China Statistical Yearbook*
*CSL*	The logarithm of the ratio of urban population to year-end household population	240	-0.523	-0.971	-0.110	0.184	*China Statistical Yearbook*
*TEC*	The logarithm of Technology Market Turnover as a share of real GDP	240	-0.327	-3.952	3.762	1.370	*China Science and Technology Statistical Yearbook*

### Data sources

This paper uses panel data from 30 provinces, municipalities directly under the Central Government and autonomous regions of China (excluding Tibet Autonomous Region) from 2013 to 2020 and processes the data accordingly, using Stata16, Arcgis10.2 and Geoda1.12 software for correlation analysis.(1)To facilitate the econometric analysis, all indicator data are logarithmically processed.(2)In order to eliminate the effects of inflation and price factors, GDP was deflated using the GDP deflator for the base period of 2013, and other relevant indicators using GDP were calculated from the deflated real GDP.(3)In order to ensure the completeness of the data, the missing values of very few years and regions are filled in using the index smoothing method to predict the missing values. The above data are obtained from *China Statistical Yearbook*, *China Science and Technology Statistical Yearbook* and various local statistical bulletins.

## Empirical study

### Spatial weight matrix setting

Technology linkage weight matrix: the product of the inverse of the squared geographic distance and the indicator characterizing innovation capacity (technology innovation capacity expressed as the number of patents granted per 10,000 persons), denoted as W_1_.

Technology association matrix (W_1_). Compared with the adjacency matrix and the geographic matrix, the former can only reflect the interrelationship between the geographic distances of spatial units and does not fully reflect the spatial mobility of innovation factors. This paper draws on the weight matrix construction method of Lim and Ma et al. [40, 41]. The former is a spatial matrix established to study the spatial correlation of innovation activities in American cities, and the latter is a spatial matrix established to study the spatial pattern of innovation output in China. Therefore, this paper establishes the following technology correlation matrix, which can more accurately reflect the spatial correlation of technological innovation capabilities.


$$W_{\mathrm{ij}}=\langle \begin{array}{l} \frac{Q_{i}Q_{j}}{d_{\mathrm{ij}}^{2}},\;i\neq j\\ 0,\;i=j \end{array}$$ (1)

Where the geographical distance between region *i* and *j* is *d*_*ij*_, and *Qi* and *Q*_*j*_ denote the technological innovation capacity of region *i* and *j*, *respectively*, expressed using the average number of patents granted per 10,000 capital in each province from 2013 to 2020.

### Spatial correlation test

The premise of the spatial econometric analysis is the existence of spatial dependence on the research object, so it is necessary to test the spatial correlation of RTIC indicators. In this paper, the RTIC indicators from 2013 to 2020 are selected, and the global Moran’s I is used to test whether the RTIC is spatially correlated using the technology correlation matrix, weight matrix, and the correlation test results. As shown in Table 3, they are all significantly positive, indicating that there is a significant spatial correlation of RTIC, therefore, it is necessary to use spatial econometric regression analysis.

**Table 3:** Global Moran index results.

Time	RTIC
2013	0.242*** (0.001)
2014	0.238*** (0.001)
2015	0.227*** (0.002)
2016	0. 199*** (0.004)
2017	0. 194*** (0.005)
2018	0. 186*** (0.006)
2019	0. 183*** (0.006)
2020	0. 198*** (0.004)

Note: where***, **, and* indicate significant underwater at 1%, 5%, and 10%, respectively; corresponding *P* values are in parentheses.

### Model setting

The spatial Durbin model(SDM) contains both the spatial lagged terms of the explanatory and explanatory variables, which is general compared to the spatial lag and spatial error models. Since the digital economy is still in the stage of continuous development, and influenced by many environmental factors, the impact of the digital economy and other factors on RTIC has a certain time lag. Compared to the SDM, the DSDM will effectively solve the sequence correlation of spatial units, the unique effects of unobserved values on space and time, and the endogeneity difficulties [42], resulting in a more correct conclusion. According to Parent and LeSage [43], the DSDM has three components: spatial effect, time effect, and space-time effect. The spatio-temporal effect compares the spatial interaction between the past and present periods and may effectively quantify the spatial influence of RTIC’s sustainability.Therefore, taking into account the characteristics of sustainability of RTIC and in order to fully investigate the impact of factors other than the explanatory variables on RTIC, this paper establishes a DSDM of RTIC for empirical analysis.


$${RTIC}_{it}=\rho_{1}{RTIC}_{it-1}+\rho_{2}W{RTIC}_{it}+\rho_{3}W{RTIC}_{it-1}+{DIEC}_{it}\beta_{1}+W{DIEC}_{it}\beta_{2}+\sum X_{it}\beta_{3}+W\sum X_{it}\beta_{4}+\sigma_{it}+\mu_{it}+\varepsilon_{it}$$ (2)

As shown in Eq (2) above, where *RTIC*_it_ denotes the regional technological innovation capability, *DIEC*_it_ denotes the digital economy, *X*_it_ denotes other control variables, *WDIEC*_it_ denotes the effect of the core explanatory variable digital economy on the technological innovation capability of neighboring places, *WΣX*_it_ denotes the effect of other control variables on the technological innovation capability of neighboring places, *β*_1_ and *β*_3_ are parameters to be estimated, *β*_2_ and *β*_4_ are matrices of parameters to be estimated, *RTIC*_it−1_, *WRTIC*_it_ and *WRTIC*_it-1_ denote the time-lagged, space-lagged and spatio-temporal lag term respectively, *ρ*_1_,*ρ*_2_ and *ρ*_3_ are the time lag term coefficient, space lag term coefficient and spatio-temporal lag term coefficient of technological innovation, respectively, *W* is the spatial weight matrix, *σ_it_* is the time fixed effect, *μ_it_* is the individual fixed effect, *ε_it_* is the random disturbance term, and i and t denote province and year, respectively. If *ρ*_2_ = 0, *β*_2_ = *β*_4_ = 0,the short-term indirect effect will nonexistent. If *ρ*_2_ = *ρ*_3_ and *β*_2_ = *β*_4_ = 0,the long-term indirect effect will nonexistent.

Ignoring the interference’s spatial dependence, if any, will only result in a loss of efficiency. When the WRTICit, WDIECit and WΣXit variables are omitted, the estimator of the remaining parameter estimations loses their consistency. Furthermore, if the real data creation process is a spatial lag or geographic error model, the spatial Durbin model generates unbiased coefficient estimates.This paper conducts the LM test and the results were found to be significant. Further, LR test and Wald test were used to determine the applicability of SDM model, and the test results indicated that the SDM was the optimal model in this paper. After the Hausman test, the fixed-effects model was chosen for this paper. From the R^2^, Sigma^2^ and Log-L statistics, the difference between the three models Sigma^2^ and Log-L is not significant, and the estimated coefficients of most of the explanatory variables are more significant when time is fixed, and the decidable coefficient is 0.472, which is significantly higher than 0.107 when the individual is fixed and 0.045 when the double is fixed. Therefore, SDM with time fixed is selected for analysis, and the results of specific parameters are not shown due to space. At the same time, several variables in this paper, such as urbanization rate and expenditure share on science and education, have captured the impact of objective individual differences to different degrees. So the fixed effects in the model are more reflected at the time level, which is also an important reason for choosing the time-fixed effects model.On this basis, the SDM is extended to the DSDM, which can explain the influence of a change in one of the explanatory variables on the dependent variable, and the ML method was used for estimation. This influence can be decomposed into spatial effect and time effect, based primarily on short-term and long-term effect analysis.

### Spatial effect analysis

The coefficients of the variables in SDM and DSDM differ (see Table 4), and even the direction of influence changes, indicating that the explanatory variables listed in this paper don’t fully reflect the effect on RTIC, but it’s also influenced by other external influences, such as the situation of enterprises’ technological absorption capacity at the micro level and institutional policies at the macro level, but these indicators are difficult to collect data to quantify In contrast, the DSDM can partially express the influencing factors not listed that affect RTIC, and adding the lagged term can more effectively solve the endogeneity problem, so the DSDM analysis is a more effective econometric analysis method.

**Table 4:** Analysis and tests of static and dynamic SDM.

Variables	General Panel Model	Static SDM	Dynamic SDM	Space related items	Static SDM	Dynamic SDM
*RTIC (t- 1)*			1.318*** (0.000)			
W*RTIC(t)		-0.012 (0.929)	0.074 (0.628)			
W*RTIC(t- 1)			- 1.554*** (0.000)			
*DIEC*	-0.002 (0.991)	0.570*** (0.002)	0.344*** (0.000)	W*DIEC	-2.589*** (0.002)	7.759*** (0.000)
*PGDP*	0.339** (0.032)	0.045 (0.743)	0.267*** (0.000)	W*PGDP	0.378 (0.549)	0.950*** (0.000)
*FDIS*	0.138*** (0.005)	0.131*** (0.009)	-0.133*** (0.000)	W*FDIS	-0.004 (0.986)	-1.274*** (0.000)
*RDG*	0.354** (0.014)	0.230* (0.058)	0.149*** (0.001)	W*RDG	1.321*** (0.004)	1.204***(0.000)
*RDP*	0.010 (0.927)	0.006 (0.924)	-0.377*** (0.000)	W*RDP	-0.757** (0.040)	-1.563*** (0.000)
*GTE*	0.591*** (0.000)	1.273*** (0.000)	0.244*** (0.002)	W*GTE	1.303 (0.137)	8.334*** (0.000)
*CSL*	0.919* (0.057)	1.838*** (0.000)	-0.608*** (0.000)	W*CSL	-0.402 (0.702)	6.455*** (0.000)
*TEC*	0.020 (0.438)	0.098*** (0.000)	0.048*** (0.000)	W*TEC	0.036 (0.742)	0.119*** (0.002)
Log-likelihood		-976 2.894	-976 2.894			
Sigma^2^		0.097*** (0.000)	0.012*** (0.000)			
Observations	240	240	210			
R^2^	0.855	0.472	0.651			

Note: where ***, ** and * indicate that a variable is significant underwater at 1%, 5% and 10%, respectively; corresponding *P* values are in parentheses

The results of the DSDM with time fixed effects in Table 4 show that: (1) from the time dimension, the coefficient of the time lag term of RTIC passes the 1% significance level test, indicating that there is a significant time accumulated effect of RTIC, i.e., the higher the RTIC in the previous period, the higher the RTIC in the current period will be, which verifies hypothesis 3; (2) from the spatial dimension, the coefficient of the spatial lag term of innovation capability is positive but not significant, indicating that there is a spatial agglomeration of RTIC in technical and geographical neighborhoods but not significant; (3)from the spatial and temporal dimensions, the coefficient of the spatial and temporal lag term of RTIC is significant at -1.554, indicating that the RTIC of this province in the previous period will instead have a suppressive effect on the enhancement of RTIC in neighboring provinces.

The coefficient of the core explanatory variable DIEC is significantly positive, indicating that the development of the digital economy will promote the improvement of RTIC, which verifies hypothesis 1 and hypothesis 2 of this paper. PGDP, RDG, GTE and TEC all have significant promoting effects on the improvement of RTIC in this province, among which the level of economic development has the greatest promoting effect; FDIS, RDP and CSL have significant inhibiting effects on the development of RTIC in this province.

The direct effects include the influence of the explanatory variables on the RTIC of the region and the feedback effects of the neighboring places, which are not reflected in the results of Table 4, resulting in some deviations in the coefficient results, and need to further decompose the direct and indirect effects. In this paper, we choose DSDM, from the time dimension, we can divide the direct effects and indirect effects into short-term effects and long-term effects, which reflect the immediate effects and long-term effects of each factor on RTIC, and the specific results are shown in Table 5.

**Table 5:** Decomposition of direct and indirect effects of DSDM.

Variables	Short-term effects			Long-term effects		
	Direct	Indirect	Total	Direct	Indirect	Total
*DIEC*	0.278* (0.095)	7.436*** (0.000)	7.714*** (0.000)	5.074 (0.975)	1.205 (0.994)	6.278*** (0.000)
*PGDP*	0.258*** (0.000)	0.906*** (0.001)	1.164*** (0.000)	0.778 (0.986)	0.169 (0.997)	0.947*** (0.000)
*FDIS*	-0.122*** (0.000)	-1.224*** (0.000)	-1.346*** (0.000)	-0.947 (0.979)	-0.148 (0.997)	-1.095*** (0.000)
*RDG*	0.140*** (0.004)	1.158*** (0.000)	1.298*** (0.000)	0.931 (0.981)	0.126 (0.997)	1.057*** (0.000)
*RDP*	-0.365*** (0.000)	-1.481*** (0.000)	-1.845***(0.000)	-1.170 (0.985)	-0.332 (0.996)	-1.502*** (0.000)
*GTE*	0.162 (0.387)	8.009*** (0.000)	8.171*** (0.000)	6.133 (0.973)	0.517 (0.998)	6.651*** (0.000)
*CSL*	-0.669*** (0.001)	6.259*** (0.000)	5.590*** (0.000)	5.139 (0.945)	-0.590 (0.994)	4.549*** (0.000)
*TEC*	0.046*** (0.000)	0.108*** (0.008)	0.154*** (0.000)	0.132 (0.985)	-0.006 (0.999)	0.126*** (0.000)

Note: same as Table 4.

Short-term effects. (1) Short-term direct effects: according to the results (see Table 5), the development of the digital economy has a significant contribution to the improvement of RTIC in this province, in which every 1% increase in the level of the digital economy development will promote the improvement of local RTIC by 0.278%. Among influential effects of control variables, the coefficients of FDIS, RDP and CSL are negative, all of which significantly inhibit the RTIC’s enhancement of this province; the positive effect of GTE is not significant, and the rest of variables significantly promote the RTIC’s enhancement of this province, among which the coefficient of the TEC is only 0.046, which has a small influence effect, so the local government needs to strengthen protection of intellectual property rights, clean technology trading market environment, and give more policy protection to innovation subjects to stimulate their innovation enthusiasm, to promote the improvement of RTIC. In addition, increasing the proportion of FDIS in high-tech enterprises and reducing the proportion of investment in high-yield, high-pollution and low-innovation level enterprises may have reversed the negative effect on the improvement of RTIC; while expanding the number of scientific researchers, the training of research and experimental development personnel should be increased to further enhance the level of scientific research ability of talents, so as to improve the RTIC.

Short-term indirect effects: the development of the digital economy in the province will promote the improvement of the neighboring provinces’ RTIC. Every 1% increase in the level of the digital economy in the province will promote the neighboring provinces’ RTIC by 7.436%, indicating that the development of the digital economy in the province will spill over to neighboring provinces, and neighboring provinces will be radiated by the digital economy in the province to promote the neighboring provinces’ RTIC. Except for the level of PGDP, FDIS and RDP, the rest of the control variable indicators of the province promote the improvement of neighboring provinces’ RTIC, so we should strengthen the technical and economic exchanges with neighboring provinces, learn from the development model of neighboring provinces, combine with our reality, open up our own digital economy development path, share the dividends of the digital economy, and promote the province’s RTIC.

(3) Total short-term effects: from the overall development of China’s provinces and regions, the digital economy has a significant positive effect on the improvement of China’s RTIC. Except for FDIS and RDP, in the short term, all other variables significantly promote the improvement of China’s RTIC.

Long-term effects. Compared with the short-term effect, the long-term direct and indirect effects of each indicator variable are not significant. The impact of the digital economy development on RTIC in this province and neighboring provinces is not obvious in the long-term, probably because the digital economy is still an emerging field in China and it is still in the development period, and the mechanism of its long-term impact on innovation development cannot be accurately grasped at this stage. However, in terms of the overall effect, the direction of influence of each variable index is the same in the long term and short term, and the difference between the effect in the long term and the short term is not significant, which indicates that the role of the digital economy on the improvement of China’s RTIC tends to be stable in the long term, so the provinces need to consider the long term effect in addition to pursuing the role of the digital economy on the improvement of RTIC in the short term, and to combine the short term of DIEC, RDG, PGDP, GTE and TEC, etc., then start to turn short-term results into long-term performance, to continuously and steadily promote the improvement of RTIC and promote the high-quality development of China’s economy.

## Analysis of regional heterogeneity

For a long time, the uneven development of science-technology and economy in various regions of China has been an objective state. The development of the digital economy in numerous regions is becoming more politicized and disjointed [44]. In the eastern region, technological innovation capabilities and the level of the digital economy development are generally higher, and the spatial connections between provinces are relatively closer, while the central region is second, and the western region is sparsely populated and less spatially connected with the slower digital economy development and relatively weaker RTIC. When considering spatial effects, it is necessary to divide China’s provinces in the east, central and west, which aids in understanding the differences in the impact of the digital economy on RTIC in different regions, better controlling the overall development situation, promoting the development of the digital economy, and advancing the improvement of RTIC in China, and the results are shown in Table 6.

**Table 6:** Heterogeneity analysis of the impact of the digital economy on RTIC.

Effect	DSDM			
	Variables	Eastern	Middle	Western
	*RTIC(t-1)*	0.821*** (0.000)	0.831*** (0.000)	1.800*** (0.000)
	W*RTIC(t)	0.137 (0.376)	0.136 (0.402)	0.531** (0.018)
	W*RTIC(t-1)	-1.238*** (0.000)	-0.174 (0.262)	3.289*** (0.000)
Short-term direct effect	*DIEC*	0.526* (0.076)	1.325***(0.000)	6.294*** (0.000)
Short-term indirect effect	*DIEC*	5.753*** (0.000)	1.541** (0.026)	21.191*** (0.000)
Short-term aggregate effect	*DIEC*	6.279*** (0.000)	2.865*** (0.002)	27.485*** (0.000)
Long-term aggregate effect	*DIEC*	4.552*** (0.000)	8.589 (0.825)	-11.535*** (0.000)
	Log-likelihood	-5393.147	-8759.192 9	-1.211e+05
	Observations	77	56	77
	R^2^	0.709	0.756	0.273

Note: where***, **, and* indicate that a variable is significant at the 1%, 5%, and 10% statistical levels, respectively; corresponding p-values are in parentheses; Eastern region includes: Beijing, Tianjin, Hebei, Liaoning, Shanghai, Jiangsu, Zhejiang, Fujian, Shandong, Guangdong, and Hainan; Central region includes: Shanxi, Jilin, Heilongjiang, Anhui, Jiangxi, Henan, Hubei, and Hunan; Western region includes: Inner Mongolia, Guangxi, Chongqing, Sichuan, Guizhou, Yunnan, Shaanxi, Gansu, Qinghai, Ningxia and Xinjiang.

RTIC time lag coefficients are all significantly positive, and there are differences in coefficient sizes, with the effect on the west, where digital economy development is slow and RTIC levels are low, being the largest, with a coefficient of 1.800. The spatial lag coefficients of RTIC is all positive, but only it is significant in the west. The coefficients of spatial and temporal lags are negative in both the eastern and central regions, but the coefficient is not significant in the central region, indicating that the RTIC of the province in the eastern region in the previous period has a greater inhibitory effect on neighboring provinces, while the coefficient of spatial and temporal lags in the western region is significantly positive, indicating that the RTIC of the province in the western region in the previous period will promote the improvement of RTIC in neighboring provinces.

Since this paper focuses on the impact of the digital economy development on RTIC, only the impact coefficients of the core explanatory variable DIEC are shown, and other control variables are not shown again due to space. In terms of the short-term effects, the development of the digital economy contributes to the improvement of RTIC in each region in the short-term direct, indirect and total effects in the east, central and west. The promoting effect of the digital economy development on the RTIC of the province and neighboring provinces is greater in the western region than in the eastern and western regions. The development of the digital economy in the eastern region has a weaker role in promoting the RTIC of this province, probably because the eastern region has its own rapid technological and economic development, a large amount of technical support and innovation resources, and has been in the highland of national innovation development, so the digital economy has less effect on the improvement of RTIC in the east than in other regions. However, the digital economy in the western region has great potential for development, so the country needs to vigorously promote the construction of digital infrastructure in the western region, provide precise policy support, convey technology and talents, and tilt more resources in the western region to promote the development of innovation in the western region, thus contributing to the coordinated development of innovation in the eastern, central and western regions of China.

## Robustness tests

To verify the accuracy and robustness of the findings of this paper, the following robustness tests were conducted. Since the above empirical results show that long-term direct and indirect effects are non-existent, all the robustness results only show short-term direct and indirect effects, and the research variables only show the core variables of the digital economy indicators due to space. In this paper, we replace the indicator of TEC and use total technology market transactions to express and make logarithmic treatment, and the results are shown in the model setting (1) (see Table 7). Under the influence of force majeure factors such as the new crown epidemic, China’s economic and social development is affected to a certain extent, so this paper excludes the 2020 data for robustness testing, and the results are shown in the model setting (2) (see Table 7). It can be seen that the main explanatory variables and the size and direction of the impact of the time and space lag terms of RTIC are the same, so it shows that the research findings of this paper are relatively robust.

**Table 7:** Robustness test results of DSDM.

Variables		(1)			(2)	
		Direct effect	Indirect effect		Direct effect	Indirect effect
*RTIC (t-1)*	1.213*** (0.000)			0.936*** (0.000)		
W*RTIC(t)	0.088 (0.563)			0.074 (0.867)		
W*RTIC(t-1)	-1.347*** (0.000)			- 1.298*** (0.000)		
*DIEC*		0.345** (0.027)	6.814*** (0.000)		0.890*** (0.000)	8.069*** (0.000)
Other control variables		YES	YES		YES	YES
Log-likelihood		-8695.088			-8936.405	
Observations		210			180	
R2		0.669			0.575	

Note: same as Table 4.

## Conclusions and discussion

This paper examines the impact of the digital economy on inter-provincial RTIC in China using panel data from 30 provinces from 2013 to 2020 by building a dynamic spatial econometric model using both qualitative and quantitative analysis methods, and the main conclusions are as follows.(1)Under the DSDM analysis of the technology association matrix, the development of the digital economy plays a significant role in promoting the improvement of RTIC in these provinces, and the development of the digital economy in one province has a positive spatial spillover effect on the improvement of RTIC in neighboring provinces. (2) In the dynamic analysis, the coefficients of the time-lagged term of RTIC are significantly positive and the coefficients of the spatial-lagged term are positive but insignificant, indicating to some extent that RTIC has a time- accumulation effect and a spatial aggregation effect. In the short-term direct effects, DIEC, PGDP, RDG, GTE and TEC all have significant positive driving effects on RTIC of the provinces, while the rest of the explanatory variables show negative effects. In the short-term indirect effects, in addition to FDIS and RDP coefficient are negative, the remaining variables significantly promote the improvement of RTIC of neighboring provinces. In the long-term direct and indirect effects, due to the availability of data on indicators related to the digital economy, the impact of the variables was not significant from 2013 to 2020, although the long-term total effect of the digital economy on RTIC was significantly positive but lower than the short-term total effect, which to some extent indicates that the long-term direct and indirect effects need to be further explored.(3) There is regional heterogeneity in the effect of the digital economy on the improvement of RTIC. The short-term directs and indirect effects of the digital economy on RTIC’s enhancement are greater in the western region than in the eastern and central regions, and the digital economy in the eastern region has the smallest effect on RTIC’s enhancement. In the analysis of the time and space lag of RTIC, the time accumulation effect of RTIC in the western region is the largest, and the coefficients of spatial and temporal lag terms are also larger than those in the eastern and central regions.

This paper confirms the dynamic spatial spillover effect of regional innovation capacity. In the short term, the digital economy can help to improve not only local innovation capacity, but also neighboring innovation capacity. This conclusion not only adds the theory of the dynamic spatial spillover effect of regional innovation capacity, but also has considerable practical implications for our country’s efforts to continuously improve regional innovation capacity. The followings are the implications of the findings in this paper. (1)Integrating the digital economy with traditional industries, ramping up RTIC, and hastening industrial transformation and upgrade. Government should direct innovation topics to focus on core digital technologies of firms in high precision domains, while also encouraging traditional industries to undergo digital transformation and providing specific government subsidies during the transformation process.(2)Changing the structure of foreign capital introduction, attracting high-quality foreign capital, and improving foreign capital utilization efficiency, consequently changing the influence on RTIC. (3)Improving regional innovation capability is a systematic and long-term process that should not only focus on short-term effects, but also should investigate long-term mechanisms to boost RTIC. Accelerate the research and development of new digital technology, expand enterprise digital technology training and the cultivation of professional technical personnel, promote industry digitalization and intelligence development, and realize RTIC continuous improvement. (4)The spatial spillover effect of the digital economy and RTIC varies by region. Because the western region has greater development potential, it is necessary to improve digital infrastructure construction, increase financial support, introduce high-end talents, and improve the talent training system. Simultaneously, the eastern, central, and western regions should generate their own industrial digital transformation plans taking into account regional conditions, strengthen the exchange of digital transformation experience and innovation achievements among different regions, and constantly promote RTIC improvement in various regions.

## Supporting information

S1 Data 30 provinces. (DTA)

S2 Data Correlation analysis. (DTA)

S3 Data Digital economy (30 provinces). (DTA)

S1 File Empirical testing process. (DO)
